# Measles second dose vaccine uptake and associated factors among under-five children in Jigjiga City, Somali Region, Eastern Ethiopia: a community-based cross-sectional study

**DOI:** 10.3389/fpubh.2024.1395802

**Published:** 2024-07-30

**Authors:** Hafso Abdirahman Ibrahim, Abdi Wariyo, Elsai Mati Asefa, Abera Cheru, Arega Abebe Lonsako, Gebisa Dirirsa

**Affiliations:** ^1^School of Public Health, College of Medicine and Health Science, Jigjiga University, Jigjiga, Ethiopia; ^2^Department of Statistics, College of Natural and Computational Science, Jigjiga University, Jigjiga, Ethiopia; ^3^School of Environmental Health Science, College of Health and Medical Science, Haramaya University, Harar, Ethiopia; ^4^School of Nursing, College of Medicine and Health Sciences, Arba Minch University, Arba Minch, Ethiopia

**Keywords:** measles, MCV2, children, Somalia, Ethiopia

## Abstract

**Background:**

Measles is one of the leading causes of under-five mortality and morbidity worldwide. Although the routine service for the second dose of the measles-containing vaccine (MCV2) was introduced in Ethiopia recently, there is a paucity of evidence regarding its coverage and the factors that hinder its uptake at both the local and national levels. Thus, this study aimed to assess the uptake of MCV2 and its associated factors among children aged between 15 and 36 months old in Jigjiga City, Somali Region, Ethiopia.

**Methods:**

A community-based cross-sectional study was conducted among 429 children aged between 15 and 36 months old with their mothers/caregivers in Jigjiga City from April 1 to May 1, 2023. A multistage sampling technique was used and data were collected by using structured interviewer-administered questionnaires. The collected data were entered into Epi-data version 3.2 and analyzed in a statistical package for the social sciences (SPSS) version 26. Bivariate and multivariable logistic regression analyses were performed to identify factors associated with the uptake of the measles second dose vaccine. An adjusted odds ratio with 95% CI were reported and statistical significance was declared at *p* < 0.05.

**Results:**

The coverage of MCV2 among children aged between 15 and 36 months was 21.4% (95% CI: 17.7, 25.2). The educational status of the mother (AOR = 3.154; 95% CI: 1.68, 5.93), place of delivery (AOR = 1.90; 95% CI: 1.08, 3.25), postnatal care visits of the mother (AOR = 2.40; 95% CI: 1.37, 4.22), time taken to reach a health facility (AOR = 2.67; 95% CI: 1.28, 5.57), and knowledge about child vaccination (AOR = 2.43; 95% CI: 1.45, 4.08) were factors significantly associated with the uptake of the measles second dose vaccine.

**Conclusion:**

The coverage of MCV2 in the study area was low compared to the national immunization targets. Educational status of the mother/caregivers, place of delivery, postnatal care visits of the mother, time to reach a health facility, and knowledge about vaccination of children were significantly associated with measles second dose vaccination. The focus should be given to improving the awareness of mothers on the importance of child vaccination to improve the uptake of measles second dose vaccine and reduce the burden of measles in the region.

## Introduction

Measles is an acute respiratory illness caused by the highly contagious Morbillivirus. It primarily spreads through coughing, sneezing, and runny nose (1). Although measles can affect individuals of all ages, particularly under-five children are more susceptible to developing complications from it (2). Despite the availability of a highly effective measles vaccine since the 1960s, measles remains a leading cause of mortality and morbidity among young children worldwide (3). In 2020, there were 149,796 reported cases of measles, resulting in 60,700 deaths globally, with 77% of cases occurring in the African region, predominantly among children (4). Ethiopia reported 3,998 cases of measles in 2019 (5).

In 2011, African countries set a goal to eradicate measles by 2020 using any feasible mechanism available (6). In line with this goal, the Measles and Rubella Initiative (MRI) has developed a strategic plan to achieve high vaccine coverage and eliminate measles through routine immunization, aiming for over 80% coverage (7). The plan includes a target of 95% immunization coverage for both the first dose (MCV1) and second doses of the measles vaccine (MCV2) in each country. The World Health Organization (WHO) and the United Nations International Children’s Fund (UNICEF) recommend administering MCV1 at 9 months and MCV2 at 15–18 months as part of routine immunization (8). In Ethiopia, the second dose of measles vaccination was introduced into the routine immunization program on 11 February 2019 at 15 months of age to enhance immunity and prevent measles outbreaks (9).

Nevertheless, studies have shown that the introduction and coverage of the measles second dose vaccine vary across countries, with different combinations and forms of administration (10). According to the WHO, global MCV2 coverage increased from 15% in 2000 to 73% in 2019, but significant regional disparities remain (11). The lowest coverage was observed in the African region (36%), while the Western Pacific region had the highest coverage (94%) (12). Within the same region, countries report varying coverage rates, such as less than 50% in Kenya (13) and 62% in Burkina Faso (14). In Ethiopia, a 2019 survey conducted by the Federal Ministry of Health reported national MCV2 coverage was 47%, with significant variation between regions, including the lowest coverage in Afar and Somali (14%) and the highest coverage in Southern Nation and Nationality Peoples (65%) (15). Later, in the year 2021, the joint report by WHO and UNICEF stated that MCV2 coverage in Ethiopia reached 70.6% (16).

In this regard, given a significant disparity in MVC2 coverage, factors influencing its uptake, and challenges in eradicating measles in developing countries including Ethiopia, there remains a gap in addressing these issues. Moreover, a variety of factors have been identified to influence the uptake of the second dose of the measles vaccine and its coverage. For instance, a study in China found that higher maternal education, higher number of births, and higher socioeconomic development were associated with increased coverage of the second dose (17). Another study in Ethiopia revealed that the child’s age, Penta3 vaccination, educational level of the head of the household, and region of residence were important factors affecting the second-dose vaccination rate (18).

Accordingly, improving measles control, raising immunization awareness, and conducting research on measles elimination strategies remain the global priorities (4). Importantly, routine coverage of MCV2 plays a crucial role in achieving measles elimination in Ethiopia, as emphasized by the WHO (19). To enhance and maintain MCV2 coverage, it is essential to identify factors associated with its uptake. As a newly implemented immunization program, currently little is known about MCV2 coverage and its associated factors in Ethiopia, particularly in the Somali Region. Therefore, this study aims to assess the coverage of the measles second dose vaccine and its associated factors among children aged between 15 and 36 months old in Jigjiga City, Somali Region, Ethiopia.

## Materials and methods

### Study design and setting

A community-based cross-sectional study was conducted among children aged between 15 and 36 months old in Jigjiga City, the administrative capital of the Somali Region, located 628 km to the east of Addis Ababa the capital city of Ethiopia. Jigjiga City is composed of four districts. From four districts, the study was conducted in two districts (Qordher and Dudahidi districts). Two selected districts in Jigjiga City are composed of 15 kebeles (six urban and nine rural kebeles). The total population of the study area is 486,822, of whom 259,918 and 62,832 are women and under-five children, respectively, in 2022. There is one referral hospital, one general hospital, one primary hospital, and three health centers in the city (20). The study was conducted from April 1 to May 1, 2023.

### Populations and eligibility

All children aged between 15 and 36 months old with their mothers/caregivers in Jigjiga City were the source population, whereas all children aged between 15 and 36 months old with their mothers/caregivers residing in randomly selected kebeles of Jigjiga City during the time of data collection were our study population. Accordingly, children aged between 15 and 36 months old with their mothers/caregivers were included in this study after the mothers/caregivers provided verbal informed consent. Children whose mothers or caretakers had mental impairments were excluded from the study.

### Sample size determination and sampling techniques

The final required sample size for this study (429) was calculated using a single population proportion formula considering 12.36% percentage of MCV2 coverage (p) from Ethiopian Mini Demographic and Health Survey (EMDHS) 2019 data (21), under the assumptions of 4% margin of error, 10% non-response rate, 95% level of confidence, and 1.5 design effect. A multistage sampling technique was used to select 429 samples. First, two districts (Qordher and Dudahidi districts) were selected by a simple random sampling method out of four districts in Jigjiga City, then six kebeles (two urban and four rural) were selected by simple random sampling out of 15 kebeles (six urban and nine rural kebeles) which are found in two selected districts. After identifying the number of eligible under-five children residing in the selected kebeles, the final sample size was proportionally allocated and followed by systematic random sampling to obtain the desired sample size. To determine the sampling interval (K), the total number of households with eligible under-five children in the selected kebeles, which is equal to 5,731, was divided by the sample size (429) to give *K* = 13. To select the first household with eligible under five children, simple random sampling was used. Then every 13 households with eligible under-five children were selected to form the sample. In households with more than one eligible under-five child, a simple random sampling method was used to choose one.

### Data collection and quality control procedure

Data were collected by interviewer-based structured questionnaires and checklists. The questionnaire was initially prepared in English and translated into Af-Somali and re-translated into English to ensure consistency. The questionnaire contains the socio-demographic characteristics of the mother/caregiver and child, access to healthcare facilities and questions about the child’s immunization coverage (22). Immunization coverage was measured through a checklist, both by recording immunization uptake from the mothers/caregivers-child booklet and the mothers/caregivers history (22). Proxy (caregivers) was used to obtain information on maternal characteristics relevant to the study during the data collection in the absence of mothers. Data were collected by five BSc-degree nurses and supervised by two MSc-degree nurses.

To maintain the quality of data, 2 days of training which focused on the relevance of the study, the objective of the study, ethical issues, informed consent before the interview, and interviewing techniques were given to data collectors and supervisors. The questionnaire was translated into the local language (Af-Somali) and a pre-test was conducted on 5% of the participants 2 weeks before the actual data collection on one of the kebele (kebele 20), which was not selected for the main study to modify any ambiguity on the questionnaire. The completeness of the data was checked by supervisors on each day of activity and the necessary feedback was given to data collectors on the next morning.

### Study variables

Uptake of MCV2 was the outcome variable, it was measured through a checklist, both by recording immunization uptake from the mothers/caregivers-child booklet and the mothers/caregivers history, and coded as “1” if the children received MCV2 and “0” if the children did not receive MCV2 (22). Whereas characteristics of mothers/caregivers (residence, age of mothers, educational status, occupation, family size, and religion), characteristics of child (age of child, sex of child, and birth order of child), maternal healthcare service availability and accessibility-related factors [antenatal care service visits, number of antenatal care service visits, Tetanus Toxoid (TT) vaccine received, Place of delivery, postnatal care visits, time to reach health facility, waiting time to get service, and health extension worker (HEW) advised at home], mothers/caregivers knowledge about vaccination of children (knowledge about vaccination, knowledge about the schedule of measles vaccination, and knowledge about benefit of measles vaccination) and immunization coverage related characteristics [MCV1, Bacillus calmette-guerin (BCG), Third Dose of Oral Polio Vaccine (OPV3), Pentavalent 3, and Pneumococcal vaccine 3] were independent variables. The mother/caregivers were categorized as having good knowledge if she or he could mention at least three vaccines either by their name, by their route (site) of administration, or by which diseases they were meant for (23) The mothers/caregivers were categorized as having good knowledge about schedules of measles vaccination if she or he answered greater than the mean score of questions assessing knowledge about schedules of measles vaccination and having poor knowledge about schedules if she or he answered less than the mean score of questions assessing knowledge about schedules of measles vaccination (23) If mothers or caregivers knew and could mention at least one type of benefit of measles vaccination, they were categorized as having good knowledge about the benefits of vaccination (23).

### Data analysis

The data were entered into Epi-data version 3.2 and exported to Statistical Package for the Social Sciences (SPSS) version 26 for analysis. Descriptive analysis like frequency and percentage for categorical variables, and mean and standard deviation for normally distributed continuous variables were used to describe the characteristics of participants and the uptake of measles second dose vaccine. By using the binary logistic regression model, bivariable analysis was done to identify the relationship between each independent variable and the outcome variable. Independent variables with *p* < 0.25 were included in the multivariable analysis. Multi-collinearity was checked by using the variance inflation factor. The model fitness was checked by the Hosmer Lemeshow goodness of fit test, then, in the multivariable logistic regression analysis, adjusted odds ratio with a 95% confidence interval (CI) was reported, and variables with *p* < 0.05 were declared to be significantly associated with the uptake of measles second dose vaccine.

## Results

### Socio-demographic characteristics of the participants

A total of 429 mothers/caregiver pairs participated in this study, with a response rate of 100%. The mean age of the mothers/caregivers was 29.5 ± 5.48 years. More than half (54.3%) of the mothers/caregivers were rural dwellers, and the majority (85.1%) were Muslim. Regarding the educational status of the mothers/caregivers, the majority (65.0%) had no formal education and were housewives. More than two-thirds (69.7%) of the under-five children’s families had more than five members. Among the participating under-five children, nearly half (46.9%) were aged between 15 and 23 months, and half of them (50.1%) were male. Approximately one-third (34.3%) of the children were fourth in birth order (Table 1).

**Table 1:** Socio-demographic characteristics of mothers/caregivers and child of under-five children in Jigjiga City, Somali Region, Ethiopia, 2023 (*n* = 429).

Variables	Category	Frequency (*n*)	Percent (%)
Residence	Urban	196	45.7
	Rural	233	54.3
Age of mother (in years)	Less than 24	62	14.5
	25–34	283	66.0
	35 and above	84	19.5
Religion	Muslim	365	85.1
	Orthodox	46	10.7
	Others^*^	18	4.2
Educational status of mothers/caregivers	No formal education	279	65.0
	Primary school	88	20.5
	Secondary and above	62	14.5
Occupation status of mothers/caregivers	Housewife	279	65.0
	Merchant	58	13.5
	Employed	39	9.1
	Daily laborer	53	12.4
Total family size	<=5	130	30.3
	>5	299	69.7
Sex of child	Male	215	50.1
	Female	214	49.9
Age of child in (months)	15–23	201	46.9
	24–34	141	32.9
	≥ 35	87	20.3
Birth order of the child	First	59	13.8
	Second	129	30.0
	Third	94	21.9
	Fourth and above	147	34.3

^*^Protestant, Catholic.

### Maternal healthcare service availability, mother/caregiver accessibility, and knowledge of child vaccination

More than two-thirds (68.3%) of the mothers had antenatal care (ANC) visits, with nearly half of them (46.9%) having visited three or more times. Two-thirds (60.1%) of the mothers had not visited postnatal care (PNC). The majority (78.1%) of the mothers received tetanus vaccinations during their last pregnancy, and two-thirds (60.4%) of them gave birth in health institutions. The average time to reach a health facility was 15 min for more than half (50.1%) of the mothers/caregivers, and the average waiting time was 30 min. More than two-thirds (61.8%) of the mothers/caregivers were not advised by health extension workers (HEW) at home. Regarding mothers/caregivers’ knowledge about child vaccination, more than half (53.1%) had poor knowledge about vaccination, and approximately half (51.5%) had poor knowledge about the schedules of measles vaccination. However, three-fourths (77.9%) of the mothers/caregivers had good knowledge about the benefits of measles vaccination (Table 2).

**Table 2:** Healthcare service availability and accessibility, and knowledge about vaccination of under-five children related factors of mother/caregivers in Jigjiga City, Somali Region, Ethiopia, 2023 (*n* = 429).

Variables	Category	Frequency (*n*)	Percent (%)
ANC visits	Yes	293	68.3
	No	136	31.7
Number of ANC visits	No visit	136	31.7
	1–2 visits	92	21.4
	Three or more visits	201	46.9
TT vaccine	Yes	335	78.1
	No	94	21.9
Place of delivery	Home	170	39.6
	Health institution	259	60.4
PNC visits of mother	Yes	258	60.1
	No	171	39.9
Time to reach the health facility	<15 min	112	26.1
	15–30 min	215	50.1
	> 30 min	102	23.8
Waiting time to get service	<15 min	164	38.2
	15–30 min	199	46.4
	> 30 min	66	15.4
HEW advised at-home	Yes	164	38.2
	No	265	61.8
Knowledge about vaccination	Poor knowledge	228	53.1
	Good knowledge	201	46.9
Knowledge about schedules of Measles vaccination	Poor knowledge	221	51.5
	Good knowledge	208	48.5
Knowledge about the benefits of measles vaccination	Poor knowledge	95	22.1
	Good knowledge	334	77.9

### Coverage of MCV2 among under-five children

**Figure 1:**
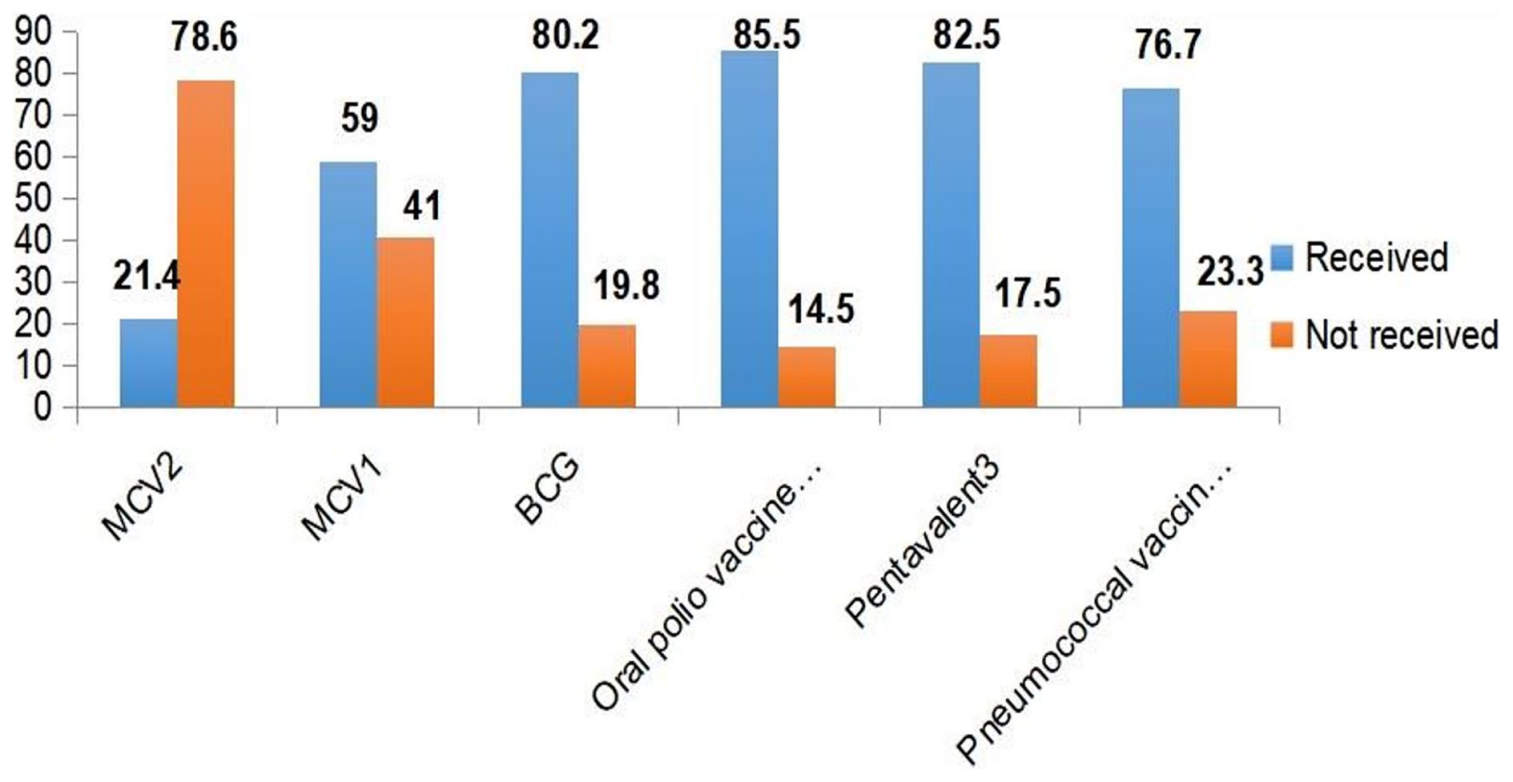
Immunization coverage among under-five children in Jigjiga City, Somali Region, Ethiopia, 2023.

Among the 429 aged between 15 and 36 months old in this study, 92 (21.4%) (95% CI: 17.7, 25.2) received the measles second dose vaccine (MCV2). More than half (59.0%) of the children received the measles first dose vaccine. The majority (80.2%) of the children received the BCG vaccine, and high coverage rates were observed for oral polio vaccine 3 (OPV3) (85.5%), Pentavalent 3 (82.5%), and pneumococcal conjugate vaccine 3 (PCV3) (76.7%) (Figure 1).

The majority of rural resident mothers/caregivers which accounts for 189(81.1%) did not receive MCV2. More than 85% of mothers or caregivers who were ≥ 35 years did not receive MCV2 whereas only 11(13.1%) received MCV2. Among children found between 24 and 34 months 39(27.7%) received MCV2 and out of children greater or equal to 35 months 19 (21.8%) received MCV2. Among total mothers delivered in health institutions about 68(26.3%) were received MCV2 were as out of mothers who visits PNC, 70(27.1%) received MCV2 (Table 3).

**Table 3:** Bivariable logistic regression of factors associated with uptake of MCV2 among under-five children in Jigjiga City, Somali Region, Ethiopia, 2023.

Variables	Categories	Status of MCV2		COR (95% CI)	*p* value
		Yes	No		
		*N* (%) (95%CI)	*N* (%) (95%CI)		
Socio-demographic of mothers/caregivers and children-related factors					
Residence	Urban	48 (24.5) (18.9, 31.0)	148 (75.5) (69.0, 81.1)	1.39 (0.88, 2.21)	0.160
	Rural	44 (18.9) (14.3, 24.5)	189 (81.1) (75.5,85.7)	1	
Age of mother/caregivers in years	< 24 years	11 (17.7) (10.0, 29.5)	51 (82.3) (70.5, 90.0)	1	
	25–34 years	70 (24.7) (20.0, 30.1)	213 (75.3) (69.9, 80.0)	1.53 (0.75, 3.08)	0.242
	≥ 35 years	11 (13.1) (7.3, 22.3)	73 (86.9) (77.7, 92.7)	0.69 (0.28, 1.73)	0.439
Educational status of mother/caregivers	No formal education	50 (17.9) (13.8, 22.9)	229 (82.1) (77.1, 86.2)	1	
	Primary (1–8)	15 (17.0) (10.5, 26.5)	73 (83.0) (73.5, 89.5)	0.94 (0.49, 1.77)	0.851
	Secondary and above	27 (43.5) (31.6, 56.3)	35 (56.5) (43.7, 68.4)	3.53 (1.96, 6.36)	0.000
Occupation status of mother/caregivers	Housewife	57 (20.4) (16.1, 25.6)	222 (79.6) (74.4, 83.9)	1	
	Merchant	14 (24.1) (14.7, 37.0)	44 (75.9) (63.0, 85.3)	1.24 (0.63, 2.42)	0.529
	Employed	9 (23.1) (12.2, 39.3)	30 (76.9) (60.7, 87.8)	1.17 (0.53, 2.60)	0.703
	Daily laborer	12 (22.6) (13.2, 36.1)	41 (77.4) (63.9, 86.8)	1.14 (0.56, 2.31)	0.716
Total family size	≤ 5 children	28 (21.5) (15.3, 29.5)	102 (78.5) (70.5 84.7)	1.00 (0.61, 1.66)	0.975
	>5 children	64 (21.4) (17.1, 26.4)	235 (78.6) (73.6, 82.9)	1	
Sex of child	Male	42 (19.5) (14.7, 25.4)	173 (80.5) (74.6, 85.3)	1	
	Female	50 (23.4) (18.1, 29.5)	164 (76.6) (70.5, 81.9)	1.26 (0.79, 1.99)	0.334
Age of child in months	15–23 months	34 (16.9) (12.3, 22.8)	167 (83.1) (77.2, 87.7)	0.73 (0.39, 1.37)	0.323
	24–34 months	39 (27.7) (20.9, 35.7)	102 (72.3) (64.3, 79.1)	1.37 (0.73, 2.57)	0.328
	≥ 35 months	19 (21.8) (14.3, 31.9)	68 (78.2) (68.1, 85.7)	1	
Healthcare service and knowledge about vaccination of under-five children-related factors					
Number of ANC visits	No visit	20 (14.7) (9.7, 21.8)	116 (85.3) (78.2, 90.3)	1	
	1–2 visits	14 (15.2) (9.2, 24.2)	78 (84.8) (75.8, 90.8)	1.04 (0.49, 2.18)	0.915
	≥ 3visits	58 (28.9) (23.0, 35.5)	143(71.1)(64.5, 77.0)	2.35 (1.34, 4.14)	0.003
TT Vaccine	Yes	68 (20.3) (16.3, 25.0)	267 (79.7) (75.0, 83.7)	0.74 (0.44, 1.27)	0.276
	No	24 (25.5) (17.7, 35.4)	70 (74.5) (64.6, 82.3)	1	
Place of delivery	Home	24 (14.1) (9.6, 20.3)	146 (85.9) (79.7, 90.4)	1	
	Health institution	68 (26.3) (21.2, 32.0)	191 (73.7) (68.0, 78.8)	2.17 (1.29, 3.62)	0.003
PNC visits of Mother	Yes	70 (27.1) (22.0, 32.9)	188 (72.9) (67.9, 78.0)	2.52 (1.49, 4.26)	0.001
	No	22 (12.9) (8.6, 18.8)	149 (87.1) (81.2, 91.0)	1	
Time to reach the health facility	≤30 min	82 (25.1) (20.7, 30.1)	245 (74.9) (69.9, 79.3)	3.08 (1.53, 6.19)	0.002
	> 30 min	10 (9.8) (5.3, 17.4)	92 (90.2) (82.6, 94.7)	1	
Waiting time to get service	< 15 min	39 (23.8) (17.8, 30.9)	125 (76.2) (69.1, 82.2)	1.40 (0.68, 2.89)	0.357
	15–30 min	41 (20.6) (15.5, 26.8)	158 (79.4) (73.2, 84.5)	1.17 (0.57, 2.38)	0.670
	> 30 min	12 (18.2) (10.5, 29.6)	54 (81.8) (70.4, 89.5)	1	
HEW advised at-home	Yes	34 (20.7) (15.2, 27.7)	130 (79.3) (72.3, 84.8)	0.93 (0.58, 1.50)	0.777
	No	58 (21.9) (17.3, 27.3)	270 (78.1) (72.7, 82.7)	1	
Knowledge about Vaccination	Poor knowledge	31 (13.6) (9.7, 18.7)	197 (86.4) (81.3, 90.3)	1	
	Good knowledge	61 (30.3) (24.4, 37.1)	140 (69.7) (62.9, 75.6)	2.77 (1.70, 4.49)	0.000
Knowledge about schedules of Measles vaccination	Poor knowledge	35 (15.8) (11.6, 21.3)	186 (84.2) (78.7, 88.4)	1	
	Good knowledge	57 (27.4) (21.7, 33.9)	151 (72.6) (66.1, 78.3)	2.01 (1.25, 3.22)	0.004
Knowledge about the benefits of measles vaccination.	Poor knowledge	22 (23.2) (15.7, 32.8)	73 (76.8) (67.2, 84.3)	1	
	Good knowledge	70 (21.0) (16.9, 25.7)	264 (79.0) (74.3, 83.1)	0.88 (0.51, 1.52)	0.645

COR, Crude odds ratio; CI, 95% Confidence interval.

### Factors associated with MCV2 uptake

In multivariable logistic regression analysis, the educational status of the mother/caregivers, place of delivery, postnatal care visits of the mother, time to reach a health facility, and knowledge about child vaccination were significantly associated with MCV2 uptake at a *p* value <0.05.

Children whose mothers/caregivers had secondary and above education levels had 3.15 times higher odds of receiving MCV2 compared to children whose mothers/caregivers had no formal education (AOR = 3.15; 95% CI: 1.68, 5.93). The odds of MCV2 utilization were increased by 88% among children whose mothers delivered in healthcare facilities compared to those born at home [AOR = 1.88; 95% CI: (1.08, 3.25)]. The odds of receiving the MCV2 vaccine were 2.4 times higher among children whose mothers attended postnatal care as compared to those whose mothers did not attend postnatal care (AOR = 2.40; 95% CI: 1.37, 4.22). The odds of receiving the MCV2 vaccine were 2.67 higher among children at a distance of less than or equal to 30 min from health facilities as compared to children at a distance of greater than 30 min from health facilities (AOR = 2.67; 95% CI: 1.28, 5.57). Moreover, the odds of receiving the MCV2 vaccine were 2.43 times higher among children whose mothers/caregivers had awareness of vaccination as compared to children whose mother/caregivers had no awareness of vaccination [AOR = 2.43; 95% CI: (1.45, 4.08)] (Table 4).

**Table 4:** Multivariable logistic regression of factors associated with uptake of MCV2 among under-five children in Jigjiga City, Somali Region, Ethiopia, 2023 (*n* = 429).

Variable	Categories	Status of MCV2		AOR (95% CI)	*p* value
		Yes (%)	No (%)		
Residence	Urban	48 (24.5)	148 (75.5)	0.76 (0.45, 1.30)	0.324
	Rural	44 (18.9)	189 (81.1)	1	
Age of mother (in years)	< 24	11 (17.7)	51 (82.3)	1	
	25–34	70 (24.7)	213 (75.3)	1.58 (0.73, 3.41)	0.250
	≥ 35	11 (13.1)	73 (86.9)	0.72 (0.27, 1.94)	0.520
Educational status of a mother	No formal education	50 (17.9)	229 (82.1)	1	
	Primary (1–8)	15 (17.0)	73 (83.0)	0.83 (0.42, 1.64)	0.595
	Secondary and above	27 (43.5)	35 (56.5)	**3.15 (1.68, 5.94)**	0.000^***^
Number of ANC visits	No visit	20 (14.7)	116 (85.3)	1	
	1–2 visits	14 (15.2)	78 (84.8)	0.95 (0.43, 2.10)	0.894
	≥ 3visits	58 (28.9)	143 (71.1)	1.53 (0.82, 2.85)	0.181
Place of delivery	Home	24 (14.1)	146 (85.9)	**1**	
	Health institution	68 (26.3)	191 (73.7)	**1.88 (1.08, 3.25)**	0.024*
PNC visits of Mother	Yes	70 (27.1)	188 (72.9)	**2.40 (1.37, 4.22)**	0.002^**^
	No	22 (12.9)	149 (87.1)	**1**	
Time to reach the health facility	≤ 30 min	82 (25.1)	245 (74.9)	**2.67 (1.28, 5.57)**	0.009**
	> 30 min	10 (9.8)	92 (90.2)	**1**	
Knowledge about Vaccination	Poor knowledge	31 (13.6)	197 (86.4)	**1**	
	Good knowledge	61 (30.3)	140 (69.7)	**2.43 (1.45, 4.08)**	0.001^**^
Knowledge about schedules of measles vaccination	Poor knowledge	35 (15.8)	186 (84.2)	1	
	Good knowledge	57 (27.4)	151 (72.6)	1.28 (1.75, 2.19)	0.371

*p* value (^*^ < 0.05, ^**^ < 0.01, ^***^ < 0.001), 1 shows the reference group of different variables, VIF = 1.92, the Hosmer-Lemeshow goodness of-fit-test (gof) (*p* value = 0.105).

## Discussion

This study aimed to determine the coverage of MCV2 and associated factors among children aged between 15 and 36 months old in Jigjiga City, Somali Region, Ethiopia. It was found that the coverage of MCV2 among under-five children in Jigjiga City was 21.4% (95% CI, 17.7, 25.2). This indicates the coverage of MCV2 in this study is too low when compared with the national immunization targets (95%) (24). This result is consistent with the study conducted in Kenya, which found a coverage of 17.9% (22). However, this finding is higher than the studies conducted in Malawi (<10%) (25) and different areas of Ethiopia such as the Mini-EDHS of 2019 (12.4%) (18), and a survey at the national level of Ethiopia on MCV2 coverage in the Somali region (14%) (15). The timing differences in vaccine rollout, as Ethiopia introduced the MCV2 vaccine in 2019, could account for these disparities, presenting challenges in terms of perception, women’s knowledge, and their willingness to be vaccinated.

On the other hand, the coverage found in Jigjiga City is lower than the national level coverage of 47% (15) and studies conducted in Switzerland (88%) (26), China (68.2%) (17), Indonesia (54%) (27), Algeria, and Cape Verde (≥95%) (25), Kenya such as Trans Nzoia County (56.2%) (28), eight Sub-Saharan African (SSA) countries (44.77%) (29), and Ethiopia such as North Shoa Zone (42.5%) (30). This discrepancy may be attributed to the unequal distribution of health facilities, differences in access to immunization programs, and population attitudes toward the value of measles vaccination.

In this study, the odds of MCV2 coverage were three times higher among children whose mother/caregiver givers had secondary education or above compared to children whose mother/caregiver had no formal education. This finding is consistent with research from China (17), eight Sub-Saharan African countries (29), Ethiopia (18), North Mecha District, West Gojjam Zone, North West Ethiopia (31) and Jabitehnan District, Northwest Ethiopia (32). Educated mothers/caregivers are more likely to possess better knowledge and understanding of the benefits of vaccination, leading to higher rates of vaccination for their children. Maternal education positively influences the practice of MCV2 utilization, recognizing its role in preventing the disease.

The odds of MCV2 utilization were increased by 88% among children whose mothers delivered in healthcare facilities compared to those born at home. This finding is supported by studies conducted in China (17), Senegal Demographic and Health Survey (33) and eight Sub-Saharan African countries (29). Mothers who give birth in healthcare facilities receive education on the importance of measles vaccination from healthcare professionals, thereby increasing compliance with recommended vaccination schedules for their children. Furthermore, the odds of MCV2 utilization were two times higher among children born to mothers who attended postnatal care compared to those whose mothers did not attend postnatal care. This finding was similar with the study conducted in Southeast Bale Zone, Oromia Region, Ethiopia (34), Gambela Region, Southwest Ethiopia (23), at Mizan Aman town, Bench Maji zone, Southwest Ethiopia (35) and research from eight Sub-Saharan African countries (29). Women who access postnatal care receive counseling and health education regarding the need for MCV2 for their children. Additionally, postnatal care visits often involve scheduling future appointments for both the mother and the child. These scheduled visits serve as reminders for mothers to bring their children for vaccinations, including MCV2.

Moreover, the odds of MCV2 utilization were almost three times higher among mothers/caregivers who reported that distance to healthcare facilities ≤30 min compared to those who reported distances take more than 30 min. This finding is in line with research from Northwest Ethiopia (36), Hard-to-Reach Areas of Ethiopia (37), Kakamega County in Kenya (22), and eight Sub-Saharan African countries (29). Challenges related to travel costs, suitable roads, and travel phobia could contribute to missed MCV2 vaccinations among mothers living far from healthcare facilities.

Lastly, the odds of MCV2 utilization were two times higher among children whose mothers/caregivers had good knowledge of vaccination compared to those whose mothers/caregivers had poor knowledge. This finding is in agreement with previous studies conducted in Jabitehnan District, Northwest Ethiopia (32), Kakamega County in Kenya (22), and North Shoa Zone in Ethiopia (30). This might be due to knowledgeable mothers or caregivers being more likely to be aware of the recommended vaccination schedule, including the timing of MCV2 administration. They understand the significance of timely vaccination and the potential consequences of delayed or missed doses. On the other hand, knowledgeable mothers or caregivers are better equipped to make informed decisions about their children’s health, including immunizations. They may actively seek information, ask questions, and engage in discussions with healthcare providers. This empowerment enables them to advocate for their children’s vaccination needs and overcome barriers that may hinder vaccine uptake.

However, this study has certain limitations, the cross-sectional design of the study does not allow for establishing a temporal relationship between cause and effect. There might be social desirability bias related to self-reported data and some of the children had no vaccination cards, and information about vaccination status had to be limited to the mother’s verbal responses which might be liable to recall bias. Additionally, in this study, since the outcome is frequent (higher than 10%), the odds ratio estimated with logistic regression may tend to overestimate the strength of association (showing a stronger effect than in reality). Moreover, in this study service delivery factors on the supplier side like the availability of vaccines were not assessed. Therefore, further studies are needed in the future to address these limitations and obtain a more comprehensive understanding of MCV2 coverage and associated factors with a robust model which reports the prevalence ratio. Despite these limitations, the study findings add to the body of knowledge on MCV2 coverage and possible associated factors which were previously not addressed by the majority of the study especially among children between 15 to 36 months of age.

This study has also significant implications for public health in numerous ways. The finding of this study highlights the need for targeted interventions and strategies to improve vaccine coverage rates. Public health authorities can use this information to design and implement programs aimed at increasing awareness about the importance of the measles vaccine and addressing barriers to its uptake. This study also provides valuable insights into the factors associated with low vaccine uptake and public health officials can develop tailored interventions to address each specific factor. The findings of this study also underscore the need to strengthen healthcare systems to ensure effective vaccine delivery and monitoring. This can involve training healthcare providers to effectively communicate the benefits of vaccines, improving the availability and accessibility of vaccines and immunization services, and implementing robust surveillance systems to track vaccine coverage and identify areas of improvement.

## Conclusion and recommendations

The coverage of MCV2 uptake among children aged between 15 and 36 months old in this study area was too low compared to the national immunization targets (95%). Mothers/caregivers’ education level of secondary and above, delivery in healthcare facilities, attendance of postnatal care, proximity to healthcare facilities, and mother/caregivers’ good knowledge of vaccination were factors significantly associated with MCV2 uptake.

The focus should be given to improving mother/caregiver education, promoting facility-based deliveries, enhancing postnatal care services, addressing transportation challenges, and implementing educational campaigns to increase vaccination awareness.

## Data availability statement

The raw data supporting the conclusions of this article will be made available by the authors, without undue reservation.

## Ethics statement

The studies involving humans were approved by Research and Ethical Review Committee of Jigjiga University and the College of Medicine and Health Science (JJU/CMHS-RHERC/046/2015). The studies were conducted in accordance with the local legislation and institutional requirements. Written informed consent for participation in this study was provided by the participants’ legal guardians/next of kin.

## Author contributions

HI: Formal analysis, Writing – original draft, Writing – review & editing. AW: Conceptualization, Methodology, Writing – review & editing. EA: Conceptualization, Data curation, Writing – review & editing. AC: Conceptualization, Data curation, Methodology, Validation, Visualization, Writing – review & editing. AA: Conceptualization, Data curation, Writing – review & editing. GD: Conceptualization, Data curation, Validation, Visualization, Writing – review & editing.
